# CRISPR screening in hematology research: from bulk to single-cell level

**DOI:** 10.1186/s13045-023-01495-5

**Published:** 2023-10-24

**Authors:** Sarah Meyers, Sofie Demeyer, Jan Cools

**Affiliations:** 1 Center for Human Genetics, KU Leuven, Leuven, Belgium; 2 Center for Cancer Biology, VIB, Leuven, Belgium; 3 Leuvens Kanker Instituut (LKI), KU Leuven – UZ Leuven, Leuven, Belgium

**Keywords:** Leukemia, Hematology, CRISPR/Cas9, CRISPR screening, Single-cell, Perturb-seq, CROP-seq

## Abstract

The CRISPR genome editing technology has revolutionized the way gene function is studied. Genome editing can be achieved in single genes or for thousands of genes simultaneously in sensitive genetic screens. While conventional genetic screens are limited to bulk measurements of cell behavior, recent developments in single-cell technologies make it possible to combine CRISPR screening with single-cell profiling. In this way, cell behavior and gene expression can be monitored simultaneously, with the additional possibility of including data on chromatin accessibility and protein levels. Moreover, the availability of various Cas proteins leading to inactivation, activation, or other effects on gene function further broadens the scope of such screens. The integration of single-cell multi-omics approaches with CRISPR screening open the path to high-content information on the impact of genetic perturbations at single-cell resolution. Current limitations in cell throughput and data density need to be taken into consideration, but new technologies are rapidly evolving and are likely to easily overcome these limitations. In this review, we discuss the use of bulk CRISPR screening in hematology research, as well as the emergence of single-cell CRISPR screening and its added value to the field.

## Background

The discovery and development of RNA interference (RNAi) technology about two decades ago provided a new way to study gene function and perform genetic screens. However, this method to downregulate the expression of a specific gene came with a number of limitations, including highly variable knock-down efficiency [1]. The more recent development of CRISPR/Cas-mediated genome editing tools by Doudna and Charpentier [2], who were awarded the Nobel Prize in chemistry for this discovery in 2020, has provided a completely new and effective way to edit the genome directly. This genome editing can be performed in cell lines and primary cells in vitro and in vivo and has a huge application potential ranging from yeast or plant engineering to medical applications and also opened the way for many new types of genetic screens in bulk or at single-cell level.

## CRISPR/Cas9 genome editing

Genome editing is based on the Clustered Regulatory Interspaced Short Palindromic Repeats (CRISPR) technology that uses the RNA-guided endonuclease Cas9 (CRISPR-Associated protein) for sequence-specific cleavage of nucleic acids [2]. A single-guide RNA (sgRNA or gRNA) directs the Cas9 protein to a specific target site, defined by the sequence of the gRNA and flanked by a protospacer adjacent motif (PAM). Cleavage results in a double-strand break (DSB), which can either be repaired by error-prone non-homologous end-joining, which can introduce small insertions or deletions at the target locus, or by homology-directed repair (HDR) when a template sequence is provided [2]. This is referred to as the type II CRISPR/Cas9 knockout (CRISPRko) system [3], which results in efficient inactivation of the target gene via introduction of frameshift mutations.

Over the past years, many other variants of the Cas protein have been discovered or engineered, which utilize different PAM sequences, have increased on-target editing specificity, cleave RNA instead of DNA or have no nuclease activity at all. These variants include Cas13 (cleaves RNA), Cas9 nickase (makes single-strand breaks) or Cas12a (generates sticky overhangs instead of blunt ends) [4–9]. Alternative applications have been developed in which the Cas protein is fused to diverse effector domains to elicit a specific effects at the locus of interest. CRISPR interference (CRISPRi) or activation (CRISPRa) are methods for transcriptional repression or activation, respectively, through fusion of a catalytically inactive dead Cas9 (dCas9), with repressive (e.g. KRAB) or activating (e.g. VP64) effector domains. Additionally, the epigenome can be edited via fusion of Cas9 with epigenetic writers or erasers, such as histone or DNA (de)methylases or acetylases. Several other approaches have been developed to introduce specific mutations. This can be achieved by fusing dCas9 to AID (activation induced cytidine deaminase), or by prime editing, which makes use of a reverse transcriptase in combination with a prime editing gRNA (pegRNA) containing both the target site and the template for the new sequence to be introduced [10, 11]. More information about these and other CRISPR variants can be found in other excellent review articles [12–14].

## CRISPR screens

**Fig. 1:**
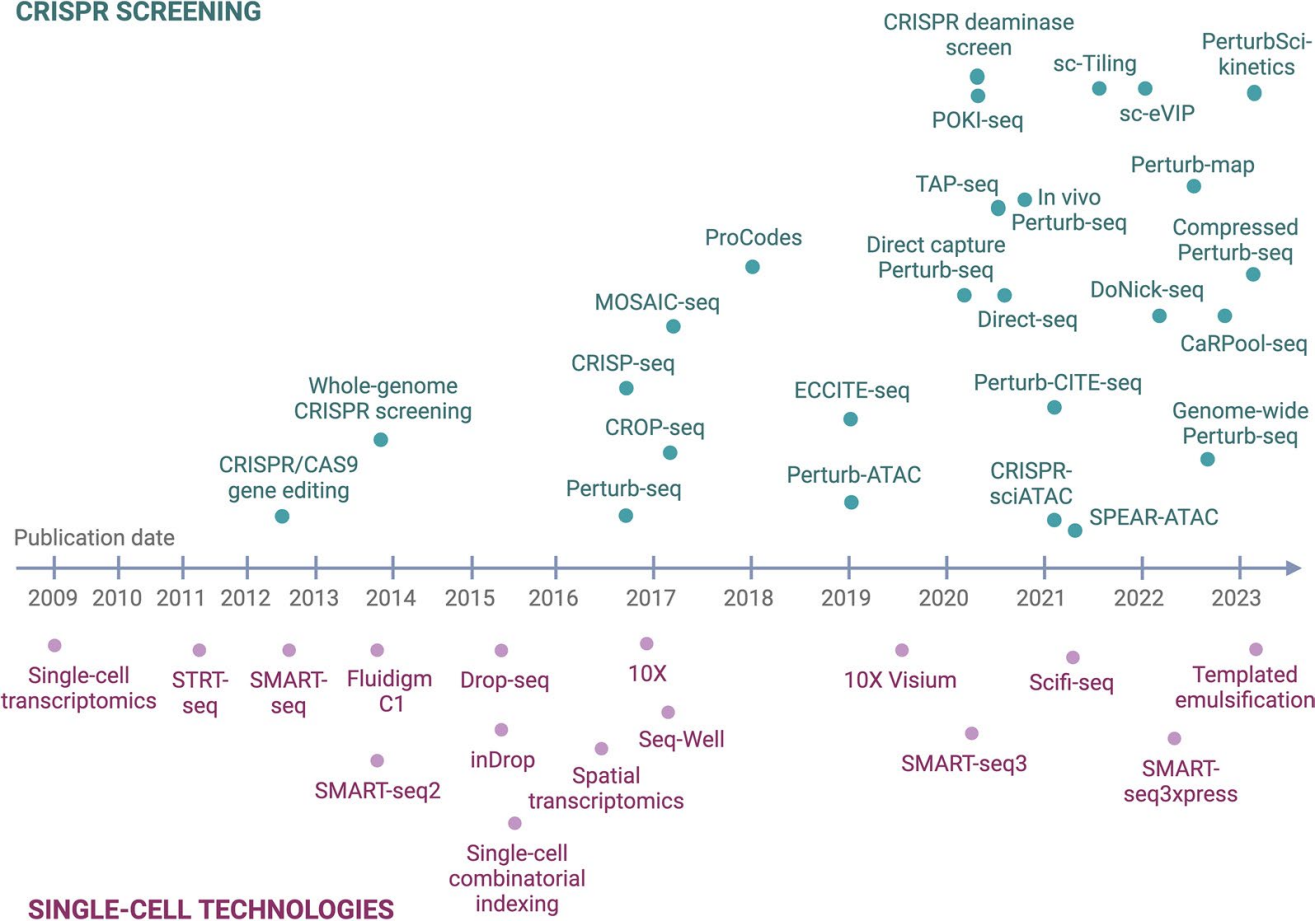
Evolution of CRISPR screening (top) and single-cell technologies (bottom) over time

**Fig. 2:**
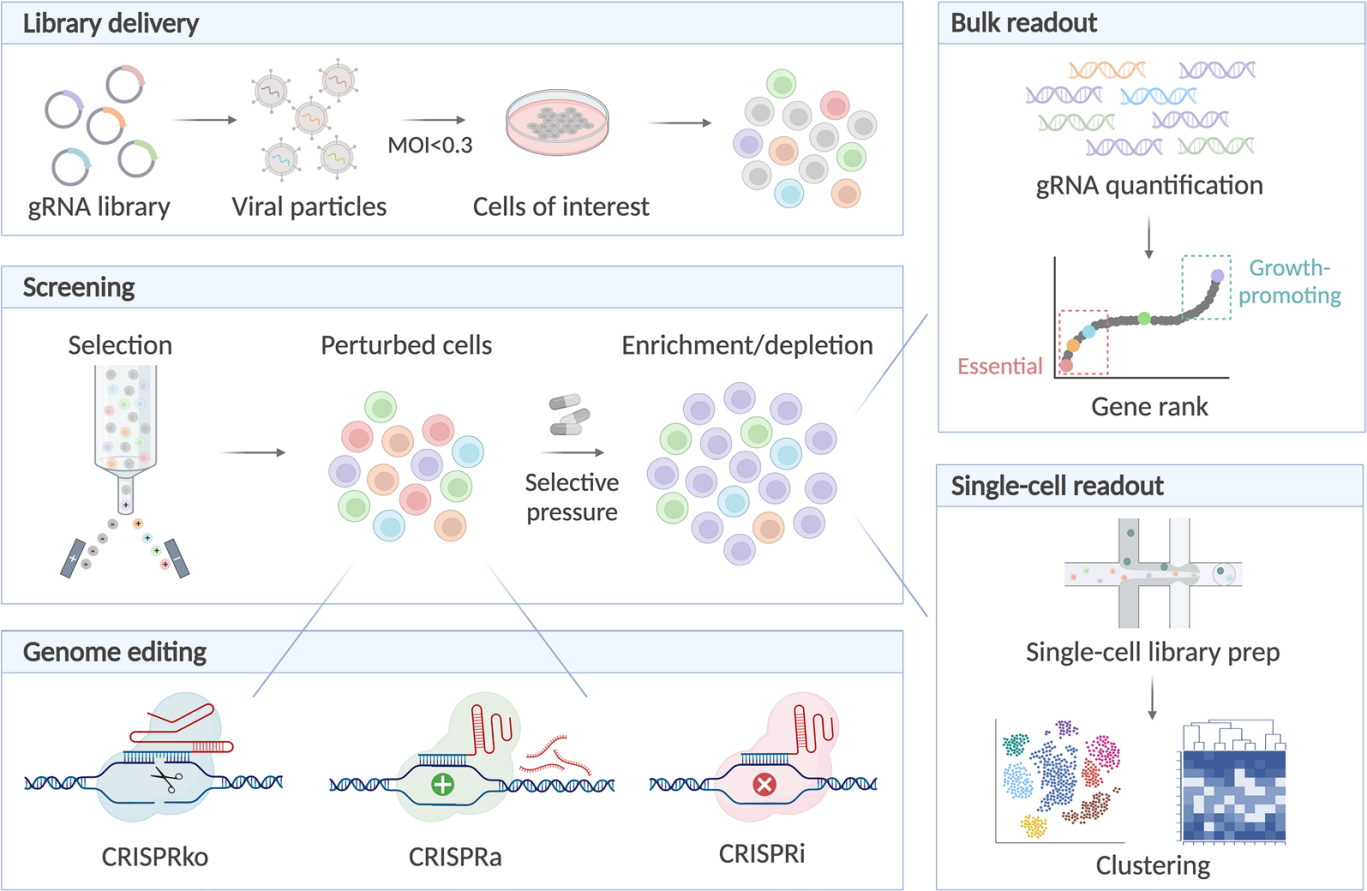
Overview of CRISPR screening with readout at bulk or single-cell level. After delivery of the gRNA library, transduced cells are enriched and undergo CRISPR editing. Perturbed cells are subjected to a selective pressure to reveal enrichment or depletion of certain sequences, which are quantified and ranked in bulk CRISPR screening. Alternatively, cells can be subjected to single-cell sequencing, revealing the transcriptomic (or multi-omic) signatures per perturbation

The CRISPR/Cas9 technology makes it possible to screen multiple perturbations simultaneously and to identify genes that are involved in specific biological processes via a forward genetics approach (Fig. 1). CRISPR screens can be performed in a pooled manner, where libraries of hundreds to thousands of gRNAs are introduced into a population of cells by viral transduction, with each cell expressing a single gRNA [15, 16]. Such screens are easily scalable to a large number of perturbations and can be applied at a genome-wide scale to interrogate thousands of loci. Ideally, libraries contain at least four gRNAs per target gene to achieve sufficient editing efficiency and are transduced at a low multiplicity of infection (MOI) (< 0.3) to ensure single-infected cells [15, 17]. Some of the editing events will have an impact on relevant processes, such as proliferation, apoptosis, migration, or drug resistance/sensitivity. Enrichment or depletion of gRNAs can be monitored by next-generation sequencing (NGS) and quantification of gRNA abundance within the cell population (Fig. 2) [18–22]. Furthermore, as in vitro screens are unable to capture the full complexity of a live organism, in vivo CRISPR screening is possible and can be used to study phenotypes in a living organism, preserving the native tissue architecture and natural microenvironment [23–25].

Since the publication of the first bulk CRISPR screens [19], many studies have used this technology in the field of leukemia research. Traditionally, the focus in this field has been on kinases, transcription factors (TF), cell cycle regulators and signaling pathways. However, recent CRISPR studies have aimed their attention toward more atypical proteins, such as RNA binding proteins (e.g. STAU1) [23], epigenetic regulators (e.g. KAT6A) [26], mitochondrial genes (e.g. MTCH2) [27] or post-translational modifiers (e.g. CMAS, SLC35A1, NANS, and GNE, involved in sialylation) [28]. Besides characterizing gene function, CRISPR screening forms a powerful tool to identify determinants of drug resistance or sensitivity, as well as synergistic drug combinations and synthetic lethalities. Oshima and colleagues have studied dependencies for the most commonly used chemotherapeutics (vincristine, 6-MP, LASP, ara-C, methotrexate, daunorubicin and maphosphamide) in acute lymphoblastic leukemia (ALL) [29]. They found common and drug-specific pathways linked to resistance. The protein phosphatase PPM1D was shown to be an essential factor for almost all of the tested drugs, while vincristine sensitivity was more dependent on mitotic factors, DNA damage repair genes influenced specifically daunorubicin response, and finally HPRT1 and SLC43A3 drove resistance to 6-MP [29]. Similarly, a study by Autry and colleagues described a genome-wide screen studying prednisolone resistance in ALL and found 14 previously unassociated genes. One of these genes was CELSR2, encoding a transmembrane receptor that upon inactivation caused BCL2 upregulation and induced sensitivity to the BCL2 inhibitor venetoclax [30]. These data illustrate that drug combinations can be used to overcome resistance development, since resistance mechanisms are largely unique to each drug, and also identify possible synergy with targeted drugs such as venetoclax.

Kinase inhibitors form another attractive group of targeted drugs, but response is often not optimal and development of resistance remains a major problem. Several studies have investigated the resistance to FLT3 inhibitors in acute myeloid leukemia (AML) and data from CRISPR screens has provided a better understanding of synergistic partners as well as potential biomarkers. In this way, PRMT5, CDK9 and DHODH were identified as synthetic lethal partners of the FLT3 inhibitor gilteritinib by inhibiting the switch to oxidative phosphorylation in FLT3-ITD AML [31]. Two studies identified loss of negative regulators of the RAS-MAPK, MTOR or WNT signaling pathways as resistance mechanisms to FLT3 inhibition, and showed promising results for the combination FLT3 inhibitors with MEK inhibitors [32, 33]. Two studies described synergy between the BCL2 inhibitor venetoclax and FLT3 inhibitor as well as HSP90 inhibitor [34, 35]. Many other co-dependencies have been discovered, such as asparaginase and BTK inhibition in ALL [36], CDK6 and MTORC1 inhibition in adult T cell leukemia/lymphoma [37], nelarabine and DUSP inhibition in AML [38], venetoclax and MCL1 inhibition in chronic lymphocytic leukemia [39] and many more.

Immunotherapy holds great potential for leukemia treatment but is often unsuccessful due to limited knowledge about the regulators and mechanisms involved. NK cells display significant anti-cancer activity, but which factors influence the susceptibility to NK cell cytotoxicity remains to be elucidated. A CRISPR screen by Zhuang et al. found that perturbation of NCR3LG1 had a protective effect, while interfering with IFN-y signaling sensitized chronic myeloid leukemia to NK cell killing [40]. Additionally, CD64 was identified as a predictive biomarker for resistance of AML to double-negative T cell therapy, while inactivation of SAGA complex members had a sensitizing effect [20]. Finally, CRISPR screens have identified modulators of CAR-T response in B cell malignancies, including the death-receptor-mediated apoptosis pathway as well as NOXA, a BCL2-family protein [41, 42].

## Single-cell CRISPR screening

A limitation to bulk CRISPR screening is that this approach can only provide information on gRNA enrichment or depletion but does not allow functional characterization of the enriched or depleted cells. An attractive alternative strategy is the combination of CRISPR screening with single-cell transcriptomic or multi-omic read-out, hereafter referred to as sc-CRISPR. Sc-CRISPR does not only reveal changes in gRNA abundance but also profiles the transcriptome (or multi-ome) of individual cells, thereby providing functional insights (Fig. 2, Table 1). In the initial sc-CRISPR approaches, originally referred to as Perturb-seq [43, 44], CRISP-seq [45], CROP-seq [46] or Mosaic-seq [47], both the mRNA and the gRNAs were sequenced, which allowed the user to link each perturbation with its transcriptional signature (Fig. 1). In addition, CRISPRi [43, 47, 48] or CRISPRa [49–51] screening can be paired with single-cell sequencing to study the consequences of gene silencing or overexpression. All these approaches generate in-depth data on the gene expression changes following perturbation and allow transcriptomic fingerprinting of genes involved in various cellular processes such as development [48, 51], immune response [44, 46, 52], differentiation [45, 53] or pathway activation [54]. An overview of possible biological applications is listed in Table 2, with specific emphasis on the studies with relevance in the field of hematology research.

**Table 1:** Overview of the technical details of the different published sc-CRISPR studies

	Cell type	gRNA capture	Perturbation type	Cas	Number of gRNAs	gRNA multiplexing	Number of cells	Delivery	Sequencing chemistry	Readout
*Transcriptomics*										
CROP-seq [46]	Jurkat	Poly-A	CRISPRko	Cas9	119	–	5798 (+ 1320 cells with NT gRNA)	Lentiviral	DROP-seq	RNA
Perturb-seq [43, 44]	K562	Barcode	CRISPRi	dCas9-KRAB	UPR epistasis screen: 9 triplet combinations UPR Perturb-seq experiment: 91	Up to 3 gRNAs in a single vector	UPR epistasis screen: 15006 UPR Perturb-seq experiment: 65337	Lentiviral	10X Genomics 3′-seq	RNA
	BMDCs, K562	Barcode	CRISPRko	Cas9	BMDC: 67 K562: 46 + 36	MIMOSCA can study interaction effects based on cells with multiple gRNAs	BMDC: 70000 K562: 104000 + 26000	Lentiviral	10X Genomics 3′-RNA-seq DROP-seq	RNA
CRISP-seq [45]	Bone marrow cells, LSK cells	Barcode	CRISPRko	Cas9	Bone marrow: 57 In vivo: 5 genes + 2 control gRNAs	Multiplexing 2 gRNAs using 2 vectors with each a different fluorophore	Bone marrow: 6144 In vivo: 2768	Lentiviral	MARS-seq	RNA
Mosaic-seq [47]	K562	Barcode	CRISPRi	dCas9-KRAB	241	High MOI	12444	Lentiviral	DROP-seq	RNA
Direct-capture Perturb-seq [74]	K562, iPSCs	gRNA-specific primer, capture sequence	CRISPRko CRISPRi CRISPRa	Cas9 dCas9-KRAB-dCas9-SunTag/scFV-VP64	K562 UPR screen: 32 iPSCs: 40 K562 interaction screen: 92 gRNA pairs K562 multiplexed screen: 87 CRISPRi, 49 CRISPRa gRNAs	gRNA pairs	K562 UPR screen: ~ 50000 iPSCs:5300 K562 interaction screen: ~ 30000 K562 multiplexed screen: ~ 30000	Lentiviral	10X Genomics 3′-seq with feature barcoding 10X Genomics 5′ seq	RNA
TAP-seq [84]	K562	Poly-A	CRISPRi	dCas9-KRAB	7055	–	231667	Lentiviral	10X Genomics 3′-seq DROP-seq	RNA
In vivo Perturb-seq [113]	Mouse embryo forebrain ventricles	Barcode	CRISPRko	Cas9	38 gRNA pairs	2 gRNAs	46770	Lentiviral injection in the forebrain	10X Genomics 3′-seq	RNA
Genome-wide Perturb-seq [136]	K562, Rpe1	Capture sequence	CRISPRi	dCas9-KRAB	11294 (K562 all expressed genes) 2291 (K562 essential genes) 2688 (Rpe1 essential genes)	2 gRNAs per vector	> 2.5 million cells	Lentiviral	10X Genomics 3′-seq with feature barcoding	RNA
Direct-seq [73]	HEK293T, Jurkat, K562	8A8G tag	CRISPRko, CRISPRa	Cas9 dCas9–VP64 MS2–p65–HSF1	12472 gRNA pairs	gRNA pairs	13435	Lentiviral	Fluidigm C1 10X Genomics 3′ and 5′-seq	RNA
Sc-Tiling [106]	MLL-AF9 murine leukemic cells	Capture sequence	CRISPRko	Cas9	602	–	4943	Lentiviral	10X Genomics 3′-seq with feature barcoding	RNA
Sc-eVIP* [111]	A549 lung cancer cells	Barcode	Coding variant overexpression	/	100 TP53 variants 101 KRAS variants	–	162314 (TP53) 150044 (KRAS)	Lentiviral	10X Genomics 3′-seq	RNA
POKI-seq [143]	Primary human T cells	Barcode	Custom HDR template	Cas9	36 knock-in templates	–	> 40000	Electroporation of Cas9 RNP with HDR templates	10X Genomics 3′-seq	RNA
Deaminase screening [108]	A375	Poly-A	base editing	BE3	420	–	13218	Lentiviral	Drop-seq	RNA
Genga et al*.* [48]	human embryonic stem cells	Poly-A	CRISPRi	dCas9-KRAB	160	–	16110	Lentiviral	10X Genomics 3′-seq	RNA
Norman et al*.* [49]	K562	Barcode	CRISPRa	dCas9-SunTag	28680 gRNA pairs	Yes, dual-gRNA vector	~ 104000	Lentiviral	10X Genomics 3′-seq	RNA
Tian et al*.* [50]	Human iPSC derived neurons	Poly-A	CRISPRi and CRISPRa	dCas9-KRAB DHFR-dCas9-VPH	CRISPRi: 374 CRISPRa: 206	–	CRISPRi: ~ 58000 CRISPRa: ~ 38000	Lentiviral	10X Genomics 3′-seq	RNA
Alda-Catalinas et al*.* [51]	Mouse embryonic stem cells	Poly-A	CRISPRa	dCas9-VP64 + MS2-p65-HSF1	475	–	203894	Lentiviral	10X Genomics 3′-seq	RNA
Belk et al*.* [52]	OT-1T cells	gRNA-specific primer	CRISPRko	Cas9	48	–	70646	Retroviral	10X Genomics V(D)J 5′ scRNA with feature barcoding	RNA
Giladi et al*.* [53]	LSK cells	Barcode	CRISPRko	Cas9	21	–	23641	Lentiviral	MARS-seq	RNA
DoNick-seq [54]	HEK293T, human intestinal crypt-like cells	Barcode	CRISPRko	Cas9 nickase	16 × 4	2 gRNA pairs	25602	Lentiviral	10X Genomics 3′-seq	RNA
PerturbSci-Kinetics [140]	HEK293	gRNA-specific primer	CRISPRi	dCas9-KRAB-MeCP2	699	–	161966	Lentiviral	Combinatorial indexing	Nascent RNA
Compressed perturb-seq [103]	THP-1	Poly-A	CRISPRko CRISPRi	Cas9 dCas9-KRAB	339	Via overloading or high MOI	Cell-pooled: 32700 guide-pooled: 24192	Lentiviral	10X Genomics 3′-seq	RNA
*Proteomics*										
Perturb-CITE-seq [132]	Melanoma cells cocultured with TILs	Poly-A	CRISPRko	Cas9	744	–	> 218000	Lentiviral	10X Genomics 3′-seq	RNA + protein
ECCITE-seq [131]	PBMC, MyLa, Sez4, NIH/3T3, K562	gRNA-specific primer	CRISPRko	Cas9	Species mixing: 20 K562: 13	–	Species mixing: 5935 K562: 4120	Lentiviral	10X Genomics 5′ V(D)J	RNA + protein + TCR/BCR
Perturb-map [123]	Kras^G12D^ p53^−/−^ lung cancer cells, 4T1	ProCode	CRISPRko	Cas9	101	–	8442439	Lentiviral	10X Genomics Visium	RNA + protein + imaging
Rothenberg et al*.* [144]	LSK cells	Capture sequence	CRISPRko	Cas9	23 gRNA pairs	gRNA pairs	~ 5000	Retroviral	10X Genomics 3′-seq with feature barcoding	RNA + protein
CaRPool-seq [104]	HEK293FT, THP1	bcgRNA	RNA silencing	RfxCas13d	29 crRNA arrays 385 crRNA arrays	CRISPR array processed by Cas13	9355 31308	Lentiviral	10X Genomics 3′-seq with feature barcoding	RNA + protein
(bee)STING-seq [107]	K562	gRNA-specific primer	CRISPRi CRISPR base editor	KRAB-dCas9-MeCP2 SpRY-Cas9-FNLS-BE3	STING-seq: 1905 beeSTING-seq: 338	–	STING-seq v1: 15285 STING-seq v2: 82339 beeSTING-seq: 39049	Lentiviral	10X Genomics 5′ seq	RNA + protein
*Epigenomics*										
Perturb-ATAC [101]	GM12878, primary human keratinocytes	Barcode	CRISPRkoCRISPRi	Cas9 dCas9-KRAB	B cells: 40 keratinocytes: 7	Yes	B cells: 2936 keratinocytes: 1356	Lentiviral	Fluidigm C1	Chromatin accessibility
CRISPR-sciATAC [124]	HEK293FT, NIH/3T3, K562	Poly-A	CRISPRko	Cas9	Species mixing: 20 Chromatin modifiers: 66 Chromatin remodeling complexes: 255	–	~ 30000	Lentiviral	Combinatorial indexing	Chromatin accessibility
SPEAR-ATAC [125]	K562, GM12878, Mcf7	gRNA-specific primer	CRISPRi	dCas9-KRAB	414	–	104592	Lentiviral	10X Genomics ATAC-seq	Chromatin accessibility

*sc-eVIP is a single-cell screen but does not involve the use of CRISPR gRNAs

**Table 2:** Summary of the biological applications of sc-CRISPR

Study	Biological application
*Hematology/leukemia*	
CROP-seq [46]	Studying TCR signaling by perturbation of transcription factors and regulators of TCR signaling in T lymphocytes
Perturb-seq [43, 44]	Characterizing the different branches of the unfolded protein response (controlled by IRE1a, ATF6 and PERK) after pharmacological UPR induction
	Transcriptional response of BMDCs to LPS stimulation Impact of perturbation of transcription factors and cell cycle regulators on myeloid cell state
CRISP-seq [45]	Studying regulators of myeloid development and identifying cell-type-specific functions
sc-Tiling [106]	Identification of functional protein domains by tiling the exons of methyltransferase DOTL1 in leukemia
POKI-seq [143]	Enhancing T cell fitness and anti-tumor immunity by pooled knock-in in the TCR locus
(bee)STING-seq [107]	Mapping GWAS loci in candidate cis-regulatory elements in erythroid cells for their impact on blood traits
CaRPool-seq [104]	Studying regulators of myeloid differentiation in the context of AML
TAP-seq [84]	Perturbation of active enhancers on chromosome 8 and 11 to map relationships between enhancers and their target genes Distinguishing the different cell types in murine bone marrow cells based on gene expression
Perturb-ATAC [101]	Regulation of keratinocyte and B cell fate by transcription factors, epigenetic regulators and non-coding RNAs
CRISPR-sciATAC [124]	Perturbation of epigenetic regulators and their impact on chromatin accessibility in myeloid leukemia cells
SPEAR-ATAC [125]	Mapping the epigenetic impact of transcription factors in myeloid differentiation
Compressed perturb-seq [103]	Analyzing the immune response of monocytic leukemia cells upon LPS stimulation
Norman et al*.* [49]	Studying regulation of erythroid differentiation
Belk et al*.* [52]	Targeting the epigenetic INO80 and BAF complexes to study T cell exhaustion of tumor infiltrating lymphocytes
Giladi et al*.* [53]	Defining the role of myeloid transcription factors in regulation of hematopoietic stem cell differentiation and progenitor cell states
Rothenberg et al. [144]	Describing the role of hematopoietic transcription factors during T cell development and commitment
*Other models*	
Direct-seq [73]	Establishing a flexible approach for gRNA capture using an 8A8G tag
Direct-capture Perturb-seq [74]	Studying genes involved in UPR pathway, cholesterol biosynthesis, DNA repair
Mosaic-seq [47]	Characterizing the contribution of enhancer activity to gene expression
sc-eVIP [111]*	Analysis of the phenotypic impact (GOF or LOF) of coding variants in TP53 and KRAS in a lung cancer model
Deaminase screening [108]	Screening for candidate resistance mutations for vemurafenib in melanoma
Perturb-CITE-seq [132]	Identification of resistance mechanisms to immune checkpoint inhibition, such as loss of certain surface markers causing immune evasion
ECCITE-seq [131]	Resolving different cell types and samples based on hashtags, transcriptomes and surface proteins, with the possibility to also capture gRNA
Perturb-map [123]	Assessing lung cancer growth and tumor microenvironment after perturbation of cytokine signaling and other immune pathways
PerturbSci-Kinetics [140]	Evaluating transcriptional dynamics regulated by genes involved in transcription initiation, chromatin remodeling, DNA replication and RNA processing
In vivo Perturb-seq [113]	Perturbing candidate risk genes for autism or neurodevelopmental disorders in the developing brain in utero
Genome-wide Perturb-seq [136]	Characterization of uncharacterized genes and describing new gene functions
DoNick-seq [54]	Studying mTORC1 regulators in conditions of amino acid starvation
Genga et al*.* [48]	identifying drivers of endoderm differentiation
Tian et al*.* [50]	Validation of hit genes from genome-wide CRISPR screen which are associated with neurodegenerative diseases
Alda-Catalinas et al.[51]	Characterizing the processes of zygotic genome activation

The list of research studies is subdivided based on their relevance in the field of hematology and/or leukemia

*sc-eVIP is a single-cell screen but does not involve the use of CRISPR gRNAs

### Single-cell technology

Single-cell sequencing is a powerful means for revealing heterogeneity within a cell population and allows the study of rare cell types which may remain concealed in bulk sequencing studies (Fig. 1). The earliest protocols for single-cell RNA-sequencing (scRNA-seq) were based on isolation of single cells in separate wells of a microwell plate, each containing lysis buffer and reagents for cDNA synthesis and barcoding (STRT-seq [55], SMART-seq [56]). These plate-based techniques allow recovery of full-length cDNA by paired-end sequencing and do not require any specialized equipment but are labor-intensive with a limited throughput. Later, the first fluidics circuits were developed by Fluidigm to separate single cells in reaction chambers on a chip [57].

The advance of high-throughput droplet-based microfluidics technology drastically increased the cell throughput via encapsulation of single cells in emulsion droplets (Drop-seq [58], inDrop [59], 10X Genomics [60]). Such technologies evolve rapidly, but currently the commercially available 10X Genomics platform is widely used as it outperforms inDrop and Drop-seq in terms of bead quality, mRNA capture efficiency and data noise [61]. Microfluidics systems have high throughput but have the disadvantage that cDNA undergoes single-end sequencing, which reduces the sensitivity and fails to detect single-nucleotide polymorphisms or isoforms. Interestingly, Scifi-seq can facilitate an even higher throughput at lower relative cost thanks to multiple rounds of combinatorial pre-indexing, pre-labeling each transcriptome with a unique combination of barcodes [62, 63]. The fact that different transcriptomes can be distinguished based on unique barcodes allows overloading of microfluidics droplets while still being able to computationally demultiplex individual transcriptomes [64]. More recently, well-based approaches are gaining popularity, where single cells are partitioned by gravitational sedimentation into nanoliter wells [65, 66]. This approach allows single cells to gently settle into a well by gravity and therefore does not require FACS sorting or microfluidics pressure, which can be harsh on the cells and may confound the transcriptome by upregulating stress-response genes. Finally, a novel scalable method was developed for single-cell encapsulation without the need for specific resources such as microfluidics or nanowells, but simply through vortexing with templated emulsification [67].

### gRNA capture approaches

A crucial consideration for sc-CRISPR is the fact that gRNAs are typically not poly-adenylated since they are transcribed from an RNA polymerase III promoter and can therefore not be detected by standard poly-A-based mRNA capture. A modified vector, that allows direct detection of either the gRNA itself or a coupled barcode sequence, is required (Table 1).

**Fig. 3:**
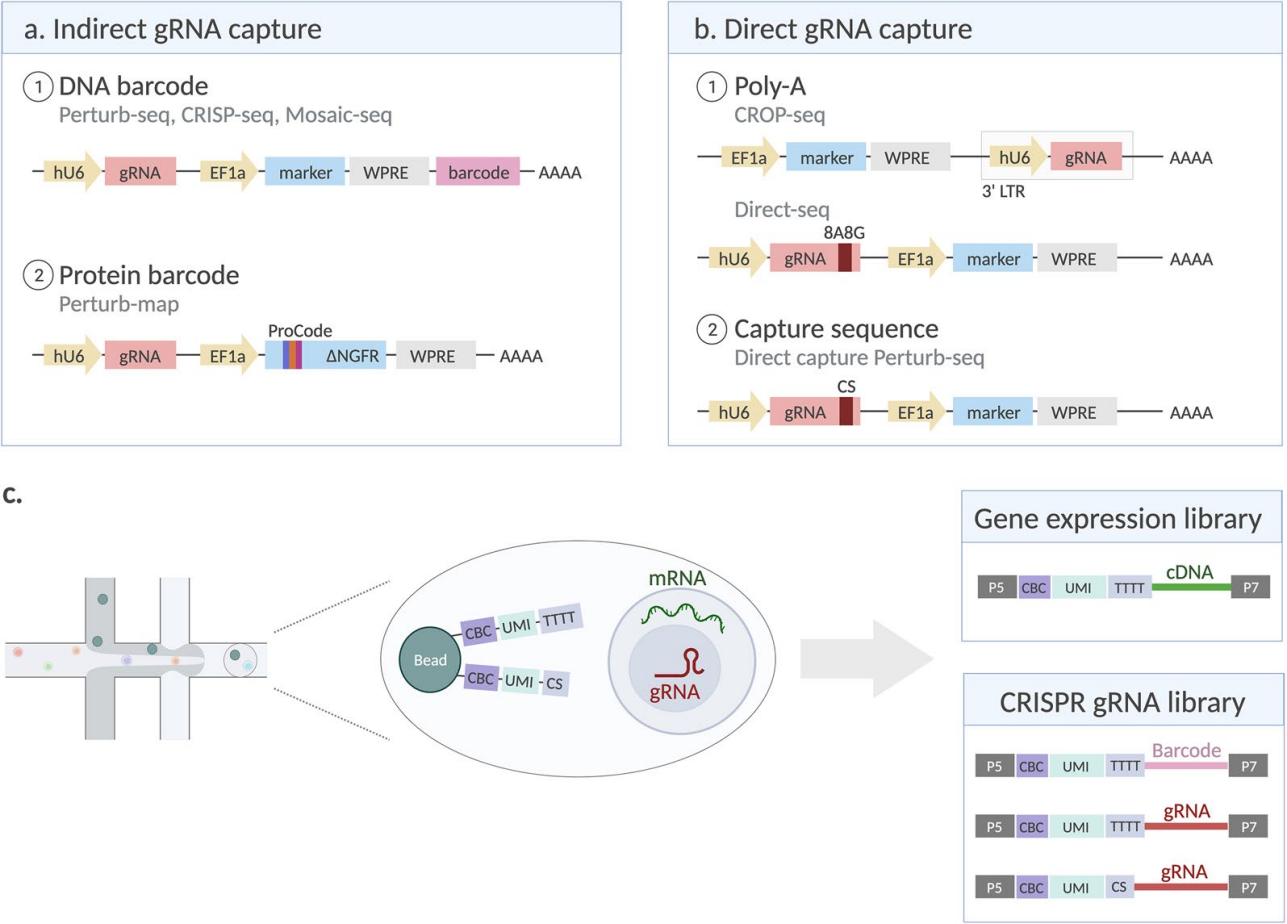
gRNA capture approaches. Due to the lack of poly-A sequence, specific measures are required for gRNA detection at single-cell level. **a** Each gRNA can be indirectly identified by a coupled DNA- or protein-based barcode. **b** Alternatively, gRNAs can be modified to include a poly-A sequence or other type of capture sequence to allow direct gRNA detection via poly-T priming or via the capture sequence. **c** After single-cell encapsulation, cDNA and gRNAs are captured by oligos on gel beads, with subsequent preparation of sequencing libraries for NGS (hU6 = human U6 promoter, EF1a = human elongation factor 1 alpha promoter, WPRE = Woodchuck Hepatitis virus posttranscriptional regulatory element, ΔNGFR = truncated nerve growth factor receptor, LTR = long terminal repeat, CS = capture sequence, CBC = cell barcode, UMI = unique molecular identifier)

Unique barcodes can be linked to a specific gRNA and preferentially amplified from the single-cell mRNA library (Fig. 3a) [43–45, 47]. However, a major issue of this approach is uncoupling of the gRNA and its barcode due to template switching during viral packaging, resulting in a lower number of good-quality cells per target gene [43, 44, 47, 68, 69]. Lentiviral recombination can be avoided by individual cloning and packaging of each construct with subsequent pooling of the virus [43]. However, such arrayed packaging is labor-intensive and poses limitations when upscaling library size. Another possible solution could be to perform lentiviral co-packaging with a low-homology carrier plasmid to prevent recombination between two gRNA copies in the pseudodiploid virion, but this comes at the cost of a severe reduction in viral titer [70, 71]. Besides DNA barcodes, combinations of antibody-detectable epitopes can be used to create protein-based barcodes (ProCodes) (Fig. 3a) [72]. Still, protein-level barcoding faces the same issues of barcode swapping as the previously described methods.

The issue of uncoupling can be eliminated by directly reading out the gRNA instead of a barcode (Fig. 3b, c). CROP-seq, as developed by Datlinger and colleagues [46], uses a modified vector where a copy of the gRNA is placed in the 3′ long terminal repeat (LTR), which gets copied to the 5′ LTR during lentiviral integration. The cassette in the 3′ LTR is transcribed by RNA polymerase II along with the other viral genes and allows poly-A based gRNA detection [46]. An alternative is Direct-seq, where an 8A8G capture sequence is incorporated in the gRNA scaffold. This sequence is a consecutive stretch of adenines mixed with guanines which can efficiently pair with a poly-T primer while still retaining sufficient editing efficiency [73]. Introducing an extra targeted amplification step to enrich for gRNA fragments in the single-cell mRNA libraries further increases the rate of gRNA assignment [68]. Alternatively, direct-capture Perturb-seq allows gRNAs to be directly sequenced alongside the transcriptome using gRNA-specific reverse transcription (RT) primers that are complementary to a capture sequence in the gRNA scaffold [74]. This method has a high capture rate and robust gRNA assignment, but efficiencies vary between different capture sequence configurations (stem loop or 3′ end) and CRISPR applications [74, 75]. 10X Genomics commercialized this method and launched their feature barcoding technology, providing gel beads which carry specific primers that can be used to detect gRNAs or other features, such as barcoded antibodies, alongside the single-cell transcriptome (Fig. 3c) [74].

### Data analysis

The main challenges in the analysis of sc-CRISPR data lie the sparsity and noise of the data, which complicates gRNA assignment as well as the analysis of the impact of each perturbation on the transcriptome. In contrast to bulk sequencing, single-cell RNA-seq has the inherent limitation that not every transcript can be recovered and especially lowly expressed genes are difficult to detect and are underrepresented. This can be partly corrected by in silico expression recovery methods, which estimate the actual gene expression based on the transcriptome profiles and the gene expression levels across cells [76].

As for all NGS data, analysis starts with mapping of the raw sequencing reads to the reference genome and the reference gRNA library. Each transcript is labeled with a cell barcode (CBC), identifying the cell of origin, and a unique molecular identifier (UMI) which enables correction for amplification artifacts. After read mapping, count matrices are generated listing all transcripts per cell, along with the assigned gRNA. Next, quality control is performed to remove low-quality cells and multiplets, based on the number of detected genes and mitochondrial transcripts, with optional regression of unwanted effects such as cell cycle and batch effects. Generally, sc-CRISPR analysis aims to estimate the impact of each perturbation on the transcriptome, to ultimately cluster the perturbations and construct complex regulatory networks.

Classic methods for differential expression analysis can be applied to compare the different perturbations, either at single-cell [77] or pseudo-bulk [78] level. The latter groups all cells with the same perturbation and determines an overall profile for this group of cells. Such pseudo-bulk profiles form a richer dataset per perturbation but lose the single-cell aspect. To make sense of the complex single-cell data, multiple algorithms have been developed, including MIMOSCA [44], Mixscape [79, 80], SCEPTRE [80], scMAGeCK [81] or MUSIC [82]. MIMOSCA uses a regularized linear model with elastic net regularization that includes technical and biological covariates [44]. Mixscape, as incorporated in the Seurat R package by the Satija lab, identifies cells with effective perturbations by comparing the signatures of cells with a gRNA versus their neighboring non-perturbed cells [79]. While this results in more reliable data from the highest quality perturbations, many cells are discarded due to the stringent filtering criteria. SCEPTRE makes use of advanced statistical methods, *i.e.* the conditional randomization test, to infer the impact of each perturbation on the transcriptome [80]. Additionally, ScMAGeCK [81] is the single-cell equivalent to the MAGeCK package [83], which is commonly used for analysis of bulk CRISPR screens. ScMAGeCK consists of two modules: robust rank aggregation (RRA) and linear regression (LR). RRA focuses on the expression of a single gene and creates a ranking based on its enrichment across the perturbations, while LR determines regulatory coefficients for all genes across all perturbations using a generalized linear model and expectation maximization. Finally, MUSIC is an integrated tool where topic modeling is used to study the biological functions associated with a particular perturbation [82].

An important limitation to the current scRNA-seq methods is the sparsity of the data and the inability to detect every possible transcript in each cell. Targeted sequencing, where only a limited set of genes is sequenced instead of the entire transcriptome, could provide a solution if expression data is only needed for a specific set of genes [74, 84]. Such enriched libraries require lower sequencing depth while providing detailed data on the expression of the most relevant genes. This drastically decreases the cost and allows sensitive screening at a larger scale, but with the disadvantage of a biased readout as it requires a priori target selection. Multiple methods exist for targeted transcript enrichment, such as multiplexed PCR [85, 86] (e.g. TAP-seq [84]), hybridization baits (e.g. HyPR-seq [87] or biotinylated hybridization baits [74]) or custom beads [88].

## New developments in single-cell CRISPR screening

### Multiplexed libraries

CRISPR screens are typically performed by perturbing a single target per cell. To achieve this, viral delivery of the gRNAs is typically performed at low MOI. However, high MOI screens may be informative to increase statistical power in case of limited cell numbers or more challenging experimental setups. In that case, multiple gRNAs can be delivered to the same cell, causing multiple perturbations simultaneously and increasing the number of cells per gRNA [89, 90]. Additionally, high MOI screens allow assessment of combinatorial perturbations and interaction effects. Since such screens generate random combinations of gRNAs, the number of combinations scales exponentially with increasing number of perturbations and an enormous number of cells would be required to cover all possible combinations.

**Fig. 4:**
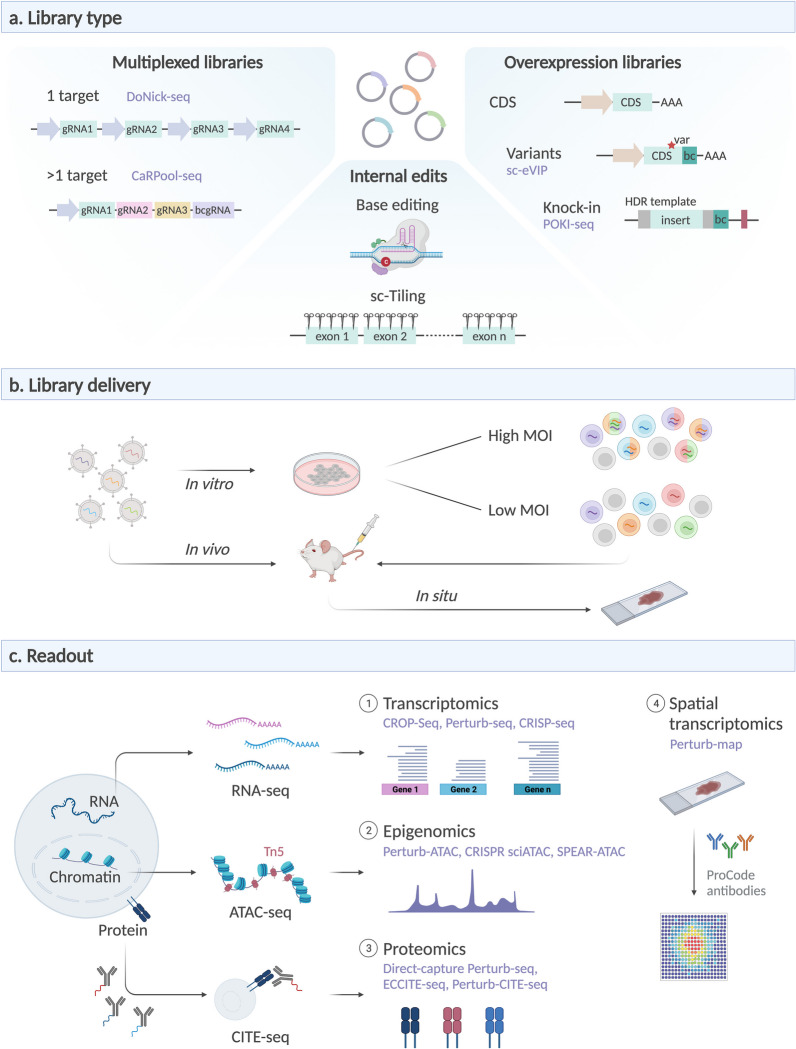
New applications of scCRISPR. **a** Different types of gRNA libraries can be used for different purposes. Multiplexed libraries target either a single gene by multiple gRNAs for highly efficient targeting, or multiple genes in a single cell to assess combinatorial effects. Base editing or tiling screens induce intragenic edits, while overexpression libraries ectopically introduce coding sequences. **b** Libraries can be delivered in vivo or in vitro, with high or low multiplicity of infection. **c** Multi-omic readouts, including transcriptomic, epigenomic or proteomic signatures, can be generated for each single cell (CDS = coding sequence, var = coding variant, HDR = homology directed repair, Tn5 = Tn5 transposase)

A specifically designed multiplexed gRNA library provides an elegant solution as this allows precise control over the combinations of gRNAs that are introduced. Multiplexed libraries carry an array of gRNAs and can be used to either target the same gene by multiple gRNAs or to target multiple different genes in the same cell (Fig. 4a). Targeting the same gene by multiple gRNAs results in increased perturbation efficiency compared to the use of a single gRNA [74, 91]. DoNick-seq studied mTORC1 pathway regulators via a double-nicking system with two pairs of gRNAs, increasing knockout efficiency by avoiding in-frame repair and reducing off-target effects [54]. Alternatively, combinatorial libraries may target multiple genes in the same cell, allowing the study of synthetic lethal interactions or genetic dependencies [49, 92–100]. Such methods have characterized the relationship between the different branches of the UPR [43] and identified synergistic and antagonistic genetic interactions regulating keratinocyte differentiation [101]. Computational tools exist for in silico prediction of promising combinations, which makes it possible to prioritize targets without having to screen every pairwise combination [102]. Additionally, compressed Perturb-seq claims to be able to computationally infer effects of individual perturbations based on composite samples containing either multiple perturbations per cell or multiple cells per emulsion droplet [103].

A different approach to multiplexing was used in CaRPool-seq. Here, the investigators used the highly efficient RNA-cleaving Cas13 for mRNA knockdown, while at the same time Cas13 was required to cleave a barcoded array of gRNAs into individual gRNAs. This way, CaRPool-seq can be used to downregulate the expression of multiple transcripts at single-cell resolution [104]. This approach was used to characterize the interactions between different regulators of myeloid differentiation in an MLL-AF9 rearranged AML model. While single perturbation of KDM1A caused enhanced expression of CD11b and a more differentiated myeloid state, combinatorial perturbation of KDM1A with either EP300 or HDAC3 led to a progenitor state or more differentiated phenotype, respectively [104]. The relevance of these data was substantiated by work showing improved response to KDM1A and HDAC inhibition in AML [105].

### Variant screening

Besides perturbing a pool of genes, there is a possibility to screen within a single gene to study functional domains or disease-relevant single-nucleotide variants (SNVs) (Fig. 4a). CRISPR tiling scanned different exons of a gene using a high-density gRNA library while simultaneously performing single-cell RNA-seq. Intragenic sc-Tiling screening revealed a novel regulatory domain of DOTL1 which impacts the methyltransferase activity as well as the response of MLL-AF9 leukemia cells to pharmacological DOTL1 targeting [106]. Moreover, CRISPR base editors can be used to introduce SNVs in a pooled manner [107, 108] and for instance found that vemurafenib resistance in melanoma is a consequence of MAP2KA and KRAS mutations [108]. Alternatively, pooled introduction of coding sequences can be employed to ectopically overexpress specific genes or introduce libraries of coding variants (Fig. 4a). A TF atlas was built this way by overexpressing all TF isoforms in embryonic stem cells and performing single-cell profiling to study changes in cell state [109]. Similarly, reprogramming of human fibroblasts was studied after introducing combinations of pro-neuronal TFs [110]. On the other hand, libraries of disease-related coding variants can be introduced via sc-eVIP, which was previously used to study TP53 and KRAS variants in a lung cancer model [111]. Finally, PoKI-seq allows pooled CRISPR knock-in screening via HDR and was used to study T cell fitness and anti-tumor activity after introducing immune-enhancing constructs [112].

### In vivo and in situ screening

Applying sc-CRISPR in vivo is an attractive strategy to assess complex biological processes and tissue-specific phenomena in the native environment of a live organism (Fig. 4b). CRISP-seq was used to study regulatory mechanisms of myeloid differentiation and immune response in vivo [45, 53]. Other in vivo studies used Perturb-seq to analyze epigenetic regulators during T cell exhaustion or neurological development in utero*,* either to assess autism risk genes or to study neuronal differentiation [113]. Even though in vivo screens with single-cell readout seem practically feasible, considerable technical challenges remain. The method and efficiency of delivery of the gRNA library can be limiting, as well as its associated cytotoxicity. In vivo studies can involve ex vivo library transduction followed by injection of the transduced cells into the animal, which can create bias as the engraftment efficiency may be low or affected by the perturbation. Many animals must be sacrificed, either to reach sufficient cell coverage per gRNA or if the cells require sequencing at multiple different timepoints. Finally, some tissues cannot readily be dissociated and require careful optimization or nuclei isolation to extract the mRNA and gRNA without significantly perturbing the transcriptome [114].

While the in vivo screens, as described above, provide transcriptome data at single-cell level, they lack spatial information. To solve this, in situ screening [115, 116] can be an interesting alternative to map gRNAs with spatial resolution using either fluorescent probes [117–120] or in situ sequencing-by-synthesis [121, 122]. A combination of in situ gRNA detection with spatially resolved single-cell RNA-seq has been described as Perturb-map, where cells were transduced by a gRNA-ProCode [72] library and subsequently injected in the target tissue of a recipient animal (Fig. 4b). After sacrifice, tissue sections were stained with ProCode-specific antibodies to spatially visualize the gRNA distribution. By integrating Perturb-map with the 10X Genomics Visium technology, sc-CRISPR can be performed with spatial resolution at near-single-cell level, retaining information on both tumor architecture and spatial context. In a mouse lung cancer model, Perturb-map characterized how each gRNA impacted the tumor itself as well as the tumor microenvironment and identified regulators of tumor growth as well as T cell infiltration [123].

### Multimodal readout of chromatin accessibility or protein

The readout for sc-CRISPR is not limited to RNA-seq but can also include epigenetic profiling or protein detection to study chromatin architecture or expression of cell surface proteins, respectively (Fig. 4c). Measuring multiple modalities in parallel adds additional layers of information to the dataset.

Epigenetic approaches (Perturb-ATAC [101], CRISPR–sciATAC [124] or SPEAR-ATAC [125]) reveal how epigenetic regulators shape the chromatin landscape. Through performing assay for transposase-accessible chromatin (ATAC-seq [126]) of single nuclei, open or closed chromatin regions can be distinguished. These recently developed ATAC-based CRISPR screens are exceptionally suited to map chromatin accessibility after perturbation of transcription factors and epigenetic regulators, which are frequently implicated in many disease contexts. Perturb-ATAC has been used to target transcription factors, epigenetic regulators and non-coding RNAs involved in B lymphocyte development. This study clustered the different perturbations based on similarities in ATAC-profiles and defined modules with specific functions during lymphoid development. Combinatorial perturbations revealed previously undescribed cooperative effects, such as IRF8 and RELA cooperating with EZH2 to repress a stem-like fate [101]. Furthermore, CRISPR sci-ATAC targeted 21 chromatin modifiers often mutated in cancer in the myeloid K562 cell line. Inactivation of EZH2 resulted in altered accessibility at HOX gene clusters, suggesting a regulatory role for EZH2 in repressing HOX gene expression [124]. Additionally, GATA1 was shown to be an essential gene in the myeloid lineage and its perturbation initially caused increased accessibility of STAT5 motifs, while increase of SPI1 motif accessibility had longer latency, highlighting time-dependent epigenetic dynamics [125]. Furthermore, activation or inactivation of non-coding cis-regulatory regions allows the identification of relationships between enhancers and the genes they regulate, thereby mapping the regulatory landscape [47, 89, 107, 127, 128].

Simultaneous transcriptome and protein sequencing was previously made possible through CITE-seq [129] or REAP-seq [130]. Prior to single-cell isolation, cells are stained with a cocktail of DNA-barcoded antibodies targeting surface proteins. These barcodes are subsequently captured via hybridization to oligos on gel beads and are compatible with both 3′ or 5′ end sequencing, enabling protein detection in each single cell [131, 132]. More importantly, these assays can now be combined with detection of CRISPR perturbations. Expanded CRISPR-compatible CITE-seq (ECCITE-seq) [79, 131] or direct-capture Perturb-seq [74] allow CRISPR screening with readout of multiple modalities in parallel, such as transcriptome, clonotype, gRNA, surface protein or cell hashing. This technology is well suited to study expression of cell surface proteins which can lead to immune evasion in patients treated with immunotherapy. PD-L1 is an inhibitory immune checkpoint molecule with great therapeutic potential and ECCITE-seq has enabled the identification of KEAP1 and NRF1 as regulators of PD-L1 expression after interferon stimulation [79]. Frangieh and colleagues applied this technology to study resistance mechanisms to immune checkpoint inhibitors in melanoma and identified loss of CD58 surface expression as a driver of immune evasion [132]. While these screens remain limited to detection of surface antigens, implementation of other methods could include detection of both extra- and intracellular (phospho)proteins and thereby enable the study of intracellular signal transduction and phosphorylation status [133–135].

### Genome-wide single-cell CRISPR screening

Only one genome-scale Perturb-seq screen has been published to date, where thousands of perturbations were profiled in over 2.5 million single cells [136]. Sc-CRISPR at such massive scale using droplet-based technology remains challenging and expensive due to the limited output of the microfluidics chips (about 10000 cells per lane). A solution to this could come from technologies that do not require chips or other special equipment. An example of this is the split-pool barcoding technology commercialized by Parse Biosciences [63], where cells are fixed, permeabilized and divided over multi-well plates in multiple rounds to label the transcriptome of each cell with a unique barcode. Such technology is scalable and first data show applications with up to 1 million cells. Alternatively, bulk genome-wide CRISPR screens can be a first step to identify interesting hits, which can subsequently be validated by focused sc-CRISPR. This enables a priori selection of potential targets whose transcriptomic signatures can subsequently be characterized at single-cell level, hence reducing cell numbers, analysis time and sequencing costs. Such focused single-cell screens have been used to better understand the unfolded protein response [43], the response of neurons to oxidative stress in the context of neurodegenerative disease [50, 137], to study regulators of T cell activation as promising targets for immunotherapy [138], as well as factors controlling viral life cycle as antiviral drug targets [139].

## Concluding remarks

CRISPR was proven a powerful tool for interrogating gene function and has greatly facilitated our understanding of biological processes and diseases. This technology has made it possible to perform pooled CRISPR screens at single-cell resolution enabling the interrogation of sets of genes to elucidate their role in disease development, drug resistance and other biological functions. The advance of sc-CRISPR does not only enable the discovery of genes with either a driving role or a tumor suppressive role but provides additional layers of high-content information on the transcriptome, proteome and/or epigenome associated with each perturbation.

The earlier publications on sc-CRISPR had a more exploratory nature and were focused on technology establishment and optimization in terms of gRNA capture and depth of read-out. These methods were subsequently used to study simple or more challenging biological questions where the limits of sc-CRISPR screens were pushed toward in vivo screens, multimodal readout, spatial resolution or even to a genome-wide level, with each their own assets and disadvantages (Tables 1, 3). These technologies remain under rapid development, with new applications such as profiling of the nascent transcriptome via PerturbSci-Kinetics, elucidating RNA dynamics [140]. Additionally, Phospho-seq enables the combination of scATAC with intracellular and intranuclear protein detection, with the possibility to integrate scRNA-seq data, combining three modalities within a single cell [141]. New single-cell technologies are being developed that do not require cell lysis for transcriptome analysis, thus keeping the cells alive and allowing temporal profiling of the same cells and studying trajectories [142]. The advent of novel CRISPR systems may further broaden the toolkit, increase on-target editing fidelity and expand the regions that can be targeted via Cas protein variants.

**Table 3:** Major technological adaptations to sc-CRISPR and their advantages and disadvantages

	Adaptation	Advantages	Disadvantages
Direct-capture Perturb-seq [74]	Direct gRNA capture via capture sequence or 5′ sequencing Targeted sequencing	Direct sequencing of the gRNA eliminates risk for barcode uncoupling Compatible with 3′ and 5′ sequencing Targeted sequencing reduces cost and increases scalability	Capture sequence may impact gRNA efficiency Requires specific resources compatible with direct gRNA capture Targeted sequencing is inherently biased
Direct-seq [73]	8A8G sequence for gRNA capture	Artificial poly-A allows poly-T-based gRNA capture Compatible with multiple different single-cell platforms Compatible with 3′ and 5′ sequencing	Requires sufficient sequencing saturation to detect gRNAs which are part of the mRNA library
DoNick-seq [54]	Cas9 nickase in combination with pairs of gRNAs	gRNA pairs enhance knockout efficiency Reduced off-target effects	More constraints for gRNA design Risk for accidental in-frame edits Not compatible with CRISPRi or CRISPRa
CaRPool-seq [104]	Cas13	Cas13 targets RNA instead of DNA Processing of CRISPR array into individual gRNAs for easy gRNA multiplexing Reduced off-target effects Cas13 protein is of smaller size than Cas9	Not compatible with CRISPRa CRISPR arrays require complex cloning strategy
Sc-Tiling [106]	CRISPR tiling	Intragenic screening Enables identification of new protein domains Multiple gRNAs close together in the same domain create a sense of redundancy and increase power	Depending on the sequence, some domains may be more difficult to target
Deaminase screening [108]	Base editing	Introduction of point mutations	Bias toward certain mutations
POKI-seq [143]	Knock-in using HDR templates	Can be applied in vivo Non-viral delivery so no integration in the host genome	Knock-in may suffer from low efficiency
(bee)STING-seq [107]	Targeting GWAS loci	Screening of non-coding regions	Screening GWAS loci tends to require large libraries with potentially little relevant hits
In vivo Perturb-seq [113]	In vivo screening	In vivo Preserves the natural microenvironment Screening circumvents the need for establishing in vivo knockout models for each target	May suffer from poor engraftment Requires optimized tissue dissociation Requires large numbers of animals
Perturb-map [123]	Spatial resolution	Preserves the spatial architecture of the tissue Allows analysis of tumor microenvironment	Does not reach actual single-cell resolution Number of perturbations is limited by the number of possible ProCode combinations
Compressed Perturb-seq [103]	Computational sample demultiplexing	Allows demultiplexing in case of multiple cells per droplet or multiple gRNAs per cell Reduced cost Requires lower cell numbers Allows analysis of interaction effects as well as individual effects	Interaction effects may complicate data analysis Computational demultiplexing might generate artifacts
TAP-seq [84]	Targeted sequencing	Requires lower sequencing depth Enables larger scale screens at a lower cost Possibility to detect lowly expressed genes	Biased Risk for poor amplification efficiency for certain amplicons
Genome-wide Perturb-seq [136]	Genome-scale	Generates extremely rich dataset	High cost in terms of reagents and sequencing Huge data analysis effort
PerturbSci-Kinetics [140]	RNA kinetics	4-thiouridine labeling distinguishes nascent RNA based on T to C conversions Allows analysis of RNA dynamics (synthesis, degradation etc.) The use of combinatorial indexing does not require specialized library preparation resources and allows scaling	Treatment with 4sU may be associated with toxicity and alter physiological cell state
Perturb-CITE-seq [132]	Proteomics	Proteomic profiling	Limited to cell surface proteins Limited number of proteins can be detected
ECCITE-seq [131]	Proteomics	Multimodal profiling: RNA, TCR, gRNA, hashing and surface protein Hashing allows sample pooling and superloading	Limited to cell surface proteins Limited number of proteins can be detected
Perturb-ATAC [101]	Epigenomics	Profiling chromatin accessibility	Low throughput No gene expression data
CRISPR-sciATAC [124]	Epigenomics	Profiling chromatin accessibility The use of combinatorial indexing does not require specialized library preparation resources and allows scaling	No gene expression data
SPEAR-ATAC [125]	Epigenomics	Profiling chromatin accessibility Improved gRNA assignment due to targeted amplification of gRNA sequences High thoughput Reduced cost	No gene expression data

This review highlighted the most recent applications of bulk CRISPR screening in hematology research and how single-cell analysis can provide added value to enhance the readout depth and elucidate the transcriptomic, epigenomic and/or proteomic signatures for each perturbation. Although these methods currently still face considerable challenges such as limited throughput and high costs, it seems plausible that single-cell screens will become an important method of CRISPR screening in the future, as it provides high-content functional characterization at single-cell resolution and can take the heterogeneity in the hematopoietic system into account.

## Data Availability

Not applicable.

## Abbreviations

ΔNGFR: Truncated nerve growth factor receptor
AID: Activation-induced cytidine deaminase
ALL: Acute lymphoblastic leukemia
AML: Acute myeloid leukemia
ATAC-seq: Assay for transposase-accessible chromatin with sequencing
beeSTING-seq: Base editing systematic targeted inhibition of noncoding GWAS loci coupled with single-cell sequencing
BMDC: Bone marrow-derived dendritic cell
CAR-T: Chimeric antigen receptor T cells
CaRPool-seq: Cas13 RNA Perturb-seq
Cas: CRISPR associated protein
CBC: Cell barcode
CDS: Coding sequence
CITE-seq: Cellular Indexing of Transcriptomes and Epitopes by Sequencing
CRISPR-sciATAC: CRISPR-based single-cell combinatorial indexing assay for transposase-accessible chromatin
CRISPR: Clustered Regularly Interspaced Short Palindromic Repeats
CRISPRa: CRISPR activation
CRISPRi: CRISPR interference
CRISPRko: CRISPR knock-out
CROP-seq: CRISPR droplet sequencing
crRNA: CRISPR RNA
CS: Capture sequence
dCAS9: Dead Cas9
DSB: Double-strand break
ECCITE-seq: Expanded CRISPR-compatible cellular indexing of transcriptomes and epitopes by sequencing
EF1a: Human elongation factor 1 alpha promoter
FACS: Fluorescence-activated cell sorting
GOF: Gain-of-function
gRNA: Guide RNA
GWAS: Genome-wide association study
HDAC: Histone deacetylase
HDR: Homology-directed repair
hU6: Human U6 promoter
HyPR-seq: Hybridization of Probes to RNA for sequencing
iPSC: Induced pluripotent stem cell
KRAB: Krüppel-associated box
LOF: Loss-of-function
LR: Linear regression
LSK cells: Lineage negative, c-Kit positive, Sca-1 positive hematopoietic stem cells
LTR: Long terminal repeat
MAPK: Mitogen-activated protein kinase
MARS-seq: Massively parallel RNA single-cell sequencing
MIMOSCA: Multiple input multiple output single-cell data analysis
MOI: Multiplicity of infection
MUSIC: Model-based understanding of single-cell CRISPR screening
NGS: Next-generation sequencing
NK cell: Natural killer cell
NT: Non-targeting
PAM: Protospacer adjacent motif
pegRNA: Prime editing gRNA
PoKI-seq: Pooled knockin sequencing
ProCode: Protein barcode
REAP-seq: RNA expression and protein sequencing assay
RNAi: RNA interference
RRA: Robust Rank Aggregation
RT: Reverse transcription
Sc-CRISPR: Single-cell CRISPR screening
Sc-eVIP: Single-cell expression-based variant impact phenotyping
SCEPTRE: Analysis of Single-CEll PerTurbation screens via conditional REsampling
Scifi-seq: Single-cell combinatorial fluidic indexing
scMAGeCK: Single-cell Model-based Analysis of Genome-wide CRISPR/Cas9 Knockout
scRNA-seq: Single-cell RNA sequencing
SMART-seq: Switching mechanism at 5′ end of RNA template sequencing
SNV: Single-nucleotide variant
SPEAR-ATAC: Single-cell perturbations with an accessibility read-out using scATAC-seq
STING-seq: Systematic targeted inhibition of noncoding GWAS loci coupled with single-cell sequencing
STRT-seq: Single-cell tagged reverse transcription sequencing
TAP-seq: Targeted Perturb-seq
TF: Transcription factor
TIL: Tumor-infiltrating lymphocytes
Tn5: Tn5 transposase
UMI: Unique molecular identifier
UPR: Unfolded protein response
WPRE: Woodchuck Hepatitis virus posttranscriptional regulatory element
